# Feasibility and early safety outcomes of robotic-assisted bariatric surgery using the Versius^®^ system: a 200-case single-center Egyptian experience

**DOI:** 10.1007/s11701-026-03209-8

**Published:** 2026-02-23

**Authors:** Mohamed D. Sarhan, M. AbdelSalam N., Mostafa Mahmoud Abdelfatah, Ehab Fathy

**Affiliations:** Faculty of Medicine - Cairo University, Cairo, Egypt

**Keywords:** Robotic-assisted surgery, Bariatric surgery, Sleeve gastrectomy, Gastric bypass, Revisional bariatric surgery, Versius^®^ system

## Abstract

Robotic assistance has been increasingly incorporated into bariatric surgery. However, data regarding newer modular robotic-assisted platforms such as the Versius^®^ Surgical Robotic System remain limited, particularly in developing healthcare settings. To evaluate the feasibility, safety, and short-term outcomes of robotic-assisted bariatric surgery (RABS) using the Versius^®^ system in a high-volume bariatric center. A retrospective analysis was conducted on 200 robotic-assisted bariatric procedures performed between December 2021 and December 2024. Procedures included primary and revisional bariatric operations. The Versius^®^ system was used as a robotic-assisted platform, with laparoscopic stapling, energy devices, and suction performed through an assistant port. Demographic data, operative metrics, complications, length of stay, and six-month postoperative outcomes were analyzed descriptively. The mean BMI was 43.85 ± 6.7 kg/m². Procedures included Sleeve Gastrectomy (*n* = 132), One-Anastomosis Gastric Bypass (*n* = 25), Roux-en-Y Gastric Bypass (*n* = 22), and Revisional Surgery (*n* = 21). Docking time decreased from 17 to 7 min, and console time stabilized after 30–40 cases. There were no conversions to open or laparoscopic surgery. The six-month morbidity rate was 1.5% (Clavien-Dindo Grade I–IIIb). RABS using the Versius^®^ platform is safe and feasible in this series, demonstrating a manageable learning curve. While the platform is adoptable in emerging healthcare settings, comparative studies are required to establish its clinical and cost advantages over standard laparoscopy.

## Key points

• Represents the first national experience with the Versius^®^ robotic platform in bariatric surgery.

• Analyzes 200 robotic-assisted bariatric procedures, including primary and revisional cases.

• Demonstrates a short learning curve with significant improvement in console and docking times.

• Confirms safety, reproducibility, and ergonomic advantages of robotic-assisted bariatric surgery.

## Introduction

The trajectory of bariatric surgery has witnessed remarkable advancements over the past few decades. Historically, bariatric procedures were performed via open surgery, involving large incisions and prolonged recovery periods. The advent of minimally invasive laparoscopic surgery revolutionized the field, offering substantial benefits such as reduced post-operative pain, shorter hospital stays, faster recovery, and improved cosmetic outcomes [1]. Laparoscopic surgery rapidly evolved into the preferred and widely accepted standard for bariatric procedures. Building upon the principles of minimally invasive surgery, robotic surgery emerged as the next technological frontier. Robotic platforms provide surgeons with enhanced dexterity, superior three-dimensional (3D) visualization, tremor filtration, and improved ergonomic positioning, potentially translating into greater precision and control during complex procedures [2]. 

The Versius^®^ Surgical Robotic System is designed to offer enhanced dexterity, precision, and control during surgical procedures. Its unique architecture, characterized by a modular design and a comparatively smaller footprint, allows for greater flexibility in operating room setup and improved access to the patient. The surgeon-controlled console provides an intuitive interface, enabling precise manipulation of articulated instruments that mimic the human wrist, facilitating complex maneuvers within confined anatomical spaces [3]. 

Robotic systems have been utilized in many surgical fields, including bariatric surgery. While the number of bariatric procedures is growing, scarce evidence is available regarding the role and outcomes of RABS using the modular Versius^®^ Surgical Robotic system specifically, rather than the more widely established systems [4]. 

This study aims to report the comprehensive experience of the first 200 cases performed using the Versius system in Egypt. It focuses on feasibility, safety, and operative efficiency, while critically examining the learning curve and short-term outcomes in a descriptive cohort.

## Methods

### Study design and setting

This study is a retrospective observational cohort analysis of a prospectively collected database. It includes 200 patients who underwent RABS between December 2021 and December 2024. No specific exclusion criteria regarding BMI, age, or comorbidities were applied, provided the patient was deemed fit for general anesthesia and bariatric surgery. However, patients who failed to complete at least 6 months of follow-up were excluded from the study.

All procedures were conducted in accordance with ethical guidelines, and institutional ethical approval was obtained before the commencement of the study (EC Ref code: N-2–2025).

### Surgical technique and Port placement

The Versius system was employed for all bariatric procedures, including primary and revisional bariatric procedures. All the procedures were performed under general anesthesia. The general setup involved positioning the patient appropriately on the operating table with a slight Trendelenburg position (head up 25 degrees). The right arm of the patient was secured by his side to allow for 2 robotic arms positioning on the right side, while his left arm was placed extended to serve as an access for the anesthesia team.

All the procedures commenced by insufflation of the abdomen, followed by the strategic placement of Versius robotic arms and ports. To ensure reproducibility, a standardized port placement strategy was adopted. Port positioning was adapted to optimize triangulation, instrument reach, and multi-quadrant access while maintaining ergonomic efficiency. The same outline was used in different procedures (Table 1) with adjustment of the vertical level (lower in bypasses and revisions, higher in sleeve gastrectomies).

**Table 1:** Outline for Port placement

Port	Size	Anatomical Location	Device/Purpose
Robotic Camera	12 mm	Midline, Supraumbilical	Optical Trocar First port placed
Robotic Arm 1	8 mm	Left Anterior Axillary Line	Robotic instrument manipulation
Robotic Arm 2	8 mm	Right Midclavicular Line	Robotic instrument manipulation
Assistant Laparoscopic Port	12 mm	Left Midclavicular Line (Below Umbilicus)	Bedside Surgeon Access Used for vessel sealing, stapling, irrigation, suction, and retraction

Routine system redocking was not required; however, limited arm repositioning was occasionally performed during multi-quadrant procedures such as gastric bypass or revisional surgery. Eventually, the need for arm repositioning diminished with experience.

At the end of every procedure, a QR code was generated by the Robotic system and scanned by the surgeon’s CMR mobile application to add the procedure to his logbook and add different metrics like Console time, Volumetric hand motion, and number of clashes per procedure to his account. Collective statistics and graphs were obtained afterwards to show important statistics and learning curve trends.

All patients’ data were collected from the hospital’s records, CMR application, and were designed in spreadsheets.

- **Primary Endpoints**: Feasibility (successful completion robotically) and Safety (intraoperative and postoperative complications).
- **Secondary Endpoints**: Operative metrics (docking/console time), Learning curve trends, and short-term (6-month) outcomes.

### Data collection

- **Preoperative data**: Demographic data, Comorbidities, Body mass index (BMI), and History of previous abdominal surgeries.
- **Hospital course data**: Operative time (Docking time and Console time), Conversion rate (laparoscopic or open), Intraoperative complications, Hospital-stay complications, Length of hospital stay (LOS).
- **Postoperative data**: morbidities encountered, Hospital readmission rate, Urgent reoperation rates, and mortality rate during the first six months post-operatively.
- **Follow-up protocol included**: (clinic visits or phone calls at 2 weeks, 1 month, 3 months, and 6 months). Cases that failed to complete 6 months of follow-up were excluded from the study.
- **Safety Grading**: Complications were graded according to the Clavien-Dindo classification.
- **Patient Reported Outcomes**: Satisfaction was recorded on a scale of 1–10 at 2 weeks. It is acknowledged that this is an unvalidated metric intended only to gauge early patient experience.

### Statistical analysis

Data were analyzed descriptively using means ± standard deviation (SD) for continuous variables and frequencies (%) for categorical variables. Trends in docking, volumetric hand motion, and console times were analyzed chronologically to evaluate the learning curve. No formal inferential statistical hypothesis testing or CUSUM analysis was performed, given the descriptive nature of this feasibility study and the heterogeneity of procedure types.

## Results

Between December 2021 and December 2024, a total of 200 robotic-assisted bariatric surgeries were performed using the Versius^®^ Surgical Robotic System Table 2.

**Table 2:** Patient demographics and baseline characteristics

Characteristic	Value (*n* = 200)
Age (years)	(Range: 29–55) Mean = 39 ± 6.2
Gender	Females: 140 (70%)
	Males: 60 (30%)
Body Mass Index (kg/m²)	(Range: 35.76–69.5) Mean = 43.85 ± 6.7
Comorbidities	
Type 2 Diabetes Mellitus	68 (34%)
Hypertension	51 (25.5%)
History of Stroke	2 cases
Surgical History (Non-Bariatric)	
Laparoscopic Cholecystectomy	15 cases
Abdominoplasty	6 cases
Midline Exploration with Consequent incisional hernia on the midline scar	1 case
History of Aortic Dissection (Bentall procedure)	1 case
Surgical History (Bariatric)	
History of Sleeve Gastrectomy	20 cases
History of Vertical Band Gastroplasty	1 case

Most cases were Sleeve Gastrectomies (SG) (132 cases). Other procedures included One Anastomosis Gastric Bypass (OAGB) (25 cases), Roux-en-Y Gastric Bypass (RYGB) (22 cases), and Revisional Bariatric Surgery (RBS) (21 cases).

Concomitant procedures included 4 cholecystectomies and 3 hiatal hernia repairs by cruroplasty.

**Fig. 1:**
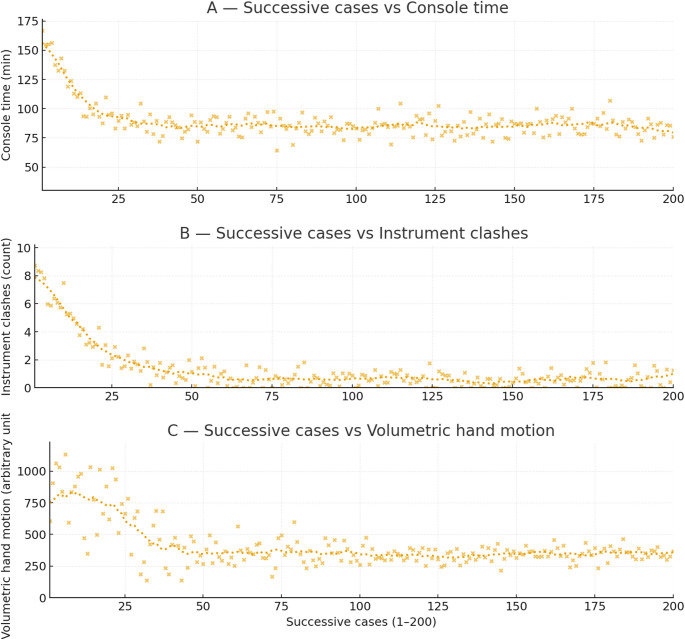
Learning curve of robotic-assisted bariatric surgery; (**A**) Console time along with case progression (Trend: Gradual decline in time across the first 30–40 cases, with subsequent stabilization). (**B**) Instrument clashes per case (higher in early cases, decreasing steadily with experience). (**C**) Volumetric hand motion per case (high variability in early procedures but stabilized with experience)

The docking time improved from an initial 17 min to a best time of 7 min. The cumulative console operating time was 276 h and 11 min, with an average duration of ~ 99 min per procedure (median 85 min), as illustrated in Fig. 1. Analysis of operative timings over the study period demonstrated a progressive reduction in console time, with stabilization of durations after the initial 30–40 cases, reflecting the impact of the learning curve.

Sleeve gastrectomy cases demonstrated the shortest console times (minimum 64 min) and required minimal arm repositioning. OAGB and RYGB procedures required longer console durations due to multi-quadrant dissection and intracorporeal anastomosis. Revisional procedures represented the longest operative times and the highest early instrument clash metrics due to adhesiolysis and altered anatomy. The only reoperation occurred following RYGB. However Operative metrics were not statistically stratified by procedure type. This is because the total number of bypass and revisional cases is relatively small compared to sleeve gastrectomy.

Analysis of system performance metrics showed a reduction in instrument clashes with increased experience, with peak values occurring in early cases and a downward trend over time (Fig. 1). Similarly, volumetric hand motion demonstrated high variability in early procedures but stabilized with experience, indicating improved ergonomic efficiency and more refined console control.

There was no need for open conversion or termination of robotic access in any of the cases. Intraoperative complications included one case of a superficial liver tear and one case of bleeding during cruroplasty.

There were no ICU admissions and no mortalities. The length of hospital stay was 1 day for all patients, except for four cases who required a 2-day stay for monitoring and optimization of medical problems. There were no complications during the hospital stay. Within the first 6 months, there was one case of wound infection, one case of thromboembolism (pulmonary and portal), and one case of hand-sewn gastrojejunostomy obstruction (one reoperation); grade (I, II, III b) Clavien-Dindo, respectively. Patient satisfaction scores at 2 weeks post-surgery ranged from 8 to 10 on a 1–10 scale.

## Discussion

This study represents the largest Egyptian case series evaluating RABS and one of the largest early-experience reports utilizing the Versius^®^ Surgical Robotic System globally.

### Feasibility and learning curve

The cumulative console operating time across the 200 procedures was 276 h and 11 min, yielding an average procedure duration of 99 min, with a median of 85 min. A key observation in this series is the significant reduction in docking and console times over the study period. The docking time improved from an initial 17 min to 7 min; this rapid decrease confirms the successful institutional mastery of the system’s setup logistics by the entire surgical team (console surgeon and bedside assistant), maximizing OR workflow and the potential case capacity of a high-volume bariatric center. Console time nearly stabilized after approximately 30–40 cases. This trend mirrors findings from other robotic platforms, where reported learning curves for an experienced laparoscopic surgeon transitioning to robotic procedures range between 20 and 40 cases [5, 6]. The mean operative duration (~ 99 min) and best recorded time for sleeve gastrectomy (64 min) are comparable to early experiences using other robotic systems [7, 8]. However, it is crucial to note that the senior surgeons in this series were experienced bariatric specialists. Therefore, the efficient learning curve is likely a combination of the system’s intuitive design and the surgeons’ preexisting proficiency, rather than the technology alone.

### Safety profile and perioperative outcomes

The absence of conversions and the low rate of major complications (0.5% reoperation rate) align with international benchmarks [9]. The inclusion of 21 revisional cases highlights the potential utility of the robotic platform in complex scenarios where anatomical distortion and adhesions make surgery challenging even in high-BMI patients (mean 43.85 kg/m²). Subjectively, the precise articulation allowed for minimal soft tissue manipulation, which may theoretically contribute to lower tissue trauma. The length of hospital stay (LOS) was 1 day for 98% of patients. This short LOS underscores the minimal invasiveness of the robotic approach and supports rapid functional recovery according to the enhanced recovery after bariatric surgery (ERABS) program [10].

High patient satisfaction scores (8–10/10), even though these data must be cautiously interpreted, being obtained using a non-validated, exploratory measure, indicate generally positive patient-perceived outcomes. It may be associated with acceptable clinical results and perceived cosmetic and recovery benefits of RABS.

### Concerns and future perspectives

Despite the positive outcomes, several ongoing debates surround RABS, including cost-effectiveness, operative time, and clinical superiority over standard laparoscopy. Although the robotic approach may initially increase operative costs, these may be offset by reduced surgeon fatigue and shorter learning curves. Furthermore, as robotic platforms become more widely available and competition increases, costs are expected to decline.

While the Versius system offers a smaller footprint and modularity, it introduces specific operational challenges distinct from single-cart robotic platforms. First, the independence of robotic arm carts requires precise preoperative planning and floor positioning to avoid external arm collisions. In our series, instrument clashes were notably higher in early cases, necessitating a dedicated learning curve for the surgical team to master port placement and docking logistics.

Also, it currently functions as a robotic-assisted tool. The reliance on a manual assistant for stapling and energy application distinguishes it from fully integrated robotic systems. This hybrid approach requires a skilled bedside assistant, which is a critical factor in the workflow efficiency observed in this study.

Future studies with longer follow-up and comparative designs (robotic vs. laparoscopic) are warranted to assess the durability of weight loss, resolution of comorbidities, and cost-benefit ratios. Additionally, objective ergonomic and workload analyses could provide insight into surgeon-centered advantages that are not directly captured in clinical metrics.

Future randomized or matched cohort studies comparing robotic versus laparoscopic bariatric procedures using the Versius system are warranted.

### Strengths and limitations

The principal strength of this study lies in its size—the largest national experience with the Versius system—and its comprehensive assessment of operative metrics, learning curve, and short-term outcomes. The inclusion of both primary and revisional procedures broadens the applicability of findings to real-world practice. However, several limitations must be acknowledged. First, the retrospective design introduces inherent bias. Also, the absence of a control laparoscopic group prevents any definitive claims regarding the superiority of the robotic approach. In addition, the follow-up period is limited to short-term safety outcomes; long-term data on weight loss and comorbidity resolution are necessary. Moreover, as a single-center experience involving expert surgeons, the results may not be immediately generalized to lower-volume centers. Finally, Operative metrics were not statistically stratified by procedure type, limiting precise quantitative comparison between SG, bypass, and revisional surgeries. This is because the total number of bypass and revisional cases is relatively small compared to sleeve gastrectomy. Patient satisfaction was measured using a non-validated exploratory scale. The study lacks ergonomic assessment and cost analysis.

## Conclusion

Robotic-assisted bariatric surgery using the Versius^®^ Surgical Robotic System was feasible and demonstrated an acceptable short-term safety profile when implemented by experienced bariatric surgeons. This report represents an early implementation and safety experience rather than a comparative effectiveness study. Prospective comparative investigations are required to definitively establish the clinical benefits and cost-effectiveness of this platform relative to standard laparoscopy.

## Data Availability

No datasets were generated or analysed during the current study.
